# Publication practices and standards: recommendations from GSK Vaccines’ author survey

**DOI:** 10.1186/1745-6215-15-446

**Published:** 2014-11-18

**Authors:** Isabelle Camby, Véronique Delpire, Laurence Rouxhet, Thomas Morel, Christine Vanderlinden, Nancy Van Driessche, Tatjana Poplazarova

**Affiliations:** GSK Vaccines, Avenue Fleming 20, 1300 Wavre, Belgium; Words and Science, Avenue du Couronnement 19, 1200 Brussels, Belgium; Health Economics and Outcomes Research group, Deloitte, Berkenlaan 8A, 1831 Brussels, Belgium; XPE Pharma and Science, Avenue Edison 19C, 1300 Wavre, Belgium

**Keywords:** Pharmaceutical industry, Good publication practice, Authorship, Survey, Policy, Transparency, Ethics

## Abstract

**Background:**

Evolving standards of good publication practice (GPP) and a survey conducted in 2009 of authors, who were investigators and researchers not employed by the company prompted changes to GSK Vaccines’ publication practices. We conducted a follow-up survey in 2012 to assess the company’s revised practices and to evaluate understanding of GPP among investigators and researchers who had previously authored at least one publication in collaboration with GSK Vaccines.

**Methods:**

The 50-question web-based survey addressed authoring practices and transparency of decision-making. Investigators and researchers (n = 1,273) who had authored at least one publication reporting on GSK Vaccines-sponsored human research since 2007, were invited to participate. Responses to 37 closed questions are presented. The remaining 13 questions were open-ended or did not concern publication practices.

**Results:**

A total of 415 external authors (32.6%) responded. International Committee of Medical Journal Editors (ICMJE) authorship criteria were clear to most respondents (78.1%); 7.7% found they were unclear. The majority of participants (86.8%) found GSK Vaccines’ authorship questionnaire a suitable tool to assess eligibility for authorship as per the ICMJE criteria. However, only 68.5% felt that the outcome of the questionnaire is communicated appropriately and 58.3% felt well informed on changes in authorship. Nearly two-thirds (62.9%) of respondents felt that having a pharmaceutical company employee as lead author makes manuscript acceptance less likely. Access to relevant data was regarded as sufficient by 78.5% of respondents. Briefing meetings before publication start, publication steering committees and core writing teams were recognized as valuable publication practices. Professional medical writing support was seen as adding value to publication development by 87.7% of participants. Most respondents agreed that manuscript discussions should start early, with 81.7% stating that they were in favor of introducing a formalized ‘author agreement’ at the publication start.

**Conclusions:**

GSK Vaccines made changes to its publication practices to ensure improved transparency and better involvement of external authors. The results of this survey suggest that these changes have been effective to a large extent. They confirm the need for effective and timely communication, as well as transparent processes for authorship and decision-making during publication development. The identified gaps in GPP will help to guide further improvements to the company’s policies on publication practices.

**Electronic supplementary material:**

The online version of this article (doi:10.1186/1745-6215-15-446) contains supplementary material, which is available to authorized users.

## Background

Authors, researchers and organizations that fund research share responsibility for developing articles in a trustworthy and ethical manner [1]. However, practices in the publication of research study results have been questioned in relation to issues such as reporting bias, inappropriate authorship and inaccurate disclosure of conflicts of interest [2–9], not only for industry-sponsored studies, but also for publicly-funded or academic trials [10–12]. This can lead to a loss of confidence in the evidence base used to inform public health decisions [13–15].

Various guidelines have been developed to standardize the reporting of research and to make an effort to ensure ethical standards are maintained in the disclosure of study findings. These include recommendations from the International Committee of Medical Journal Editors (ICMJE) for the conduct, reporting, editing and publication of scholarly work in medical journals [16]. The good publication practice (GPP) guidelines, which were published in 2003 [17] and revised by the International Society for Medical Publication Professionals (GPP2; GPP3 guidelines are in development), focus on industry-sponsored research [1]. There are also guidelines for reporting specific study types, such as the Consolidated Standards of Reporting Trials (CONSORT) for reporting randomized trials, the Strengthening the Reporting of Observational studies in Epidemiology (STROBE) guidelines, the Preferred Reporting Items for Systematic Reviews and Meta-Analyses (PRISMA) guidelines and the Consolidated Health Economic Evaluation Reporting Standards (CHEERS) guidelines for economic evaluations of health interventions [18].

Although many pharmaceutical companies have incorporated internationally-recognized best practices in their publication policies, a persistent negative view of industry-sponsored studies remains [19, 20]. Efforts have therefore been made by the industry to improve publishing practices further [10, 21–24]. In 2009, GSK Vaccines (a division of the GlaxoSmithKline group of companies) conducted a web-based survey to obtain feedback on its publication practices from 265 external authors (investigators and researchers not employed by the GlaxoSmithKline group of companies) who had collaborated with the company on at least one publication. Based on the survey’s findings and evolving international recommendations for best publication practices, GSK Vaccines introduced a number of initiatives to improve transparency and ensure better involvement of external authors in the decision-making processes for manuscript development from an early stage [23]. Measures were also put in place to increase understanding of publication standards, including open discussions of responsibilities, authorship and journal selection, and the introduction of an authorship questionnaire to determine compliance with ICMJE criteria (see Additional file 1) [25]. Company policy on public disclosure of clinical research includes a ban on ‘ghostwriting’ of journal manuscripts and abstracts, with a requirement that any medical writers who are employees or directly contracted to the company are either named as authors when eligible, or included in the acknowledgements section of manuscripts [23]. The primary author for a paper must actively participate in the drafting process, lead content development and work closely with co-authors in agreeing the final version. A publications coordinator provides logistical and editorial support to facilitate communication among authors and improve the quality of study reporting with good publication practice guidance. Publication steering committees (PSCs) and core writing teams may also be formed. A web-based file-sharing system (Datavision™; Envision Technology Solutions, Glastonbury, United Kingdom) permits centralized documentation and improves transparency in publication practices among users.

In 2012, GSK Vaccines invited nearly 1,300 external authors working with the company worldwide to participate in a second web-based survey. As follow-up to the 2009 survey, this had the objective of evaluating feedback on the usefulness of practices employed to coordinate and facilitate the development of publications. Another objective was to gain insight into authors’ understanding of key concepts in publication development relating to authorship, opinions on the transparency of publication practices and decision-making, as well as general perceptions of the publication of industry-sponsored research.

## Methods

### The survey questionnaire

The web-based survey was developed and managed by Deloitte’s health economics department (Brussels, Belgium) on behalf of GSK Vaccines. The initial proposal document for conduct of the study can be provided upon request. No incentive was offered to potential participants to take part in the survey. The survey questionnaire was first piloted within GSK Vaccines. The final version of the questionnaire was developed by Deloitte based on feedback received from participants of the test survey.

The survey included 50 questions in English that did not require more than 15 minutes to complete and focused on the main changes that had been implemented in the company’s publication practices over the previous two years (survey questionnaire is provided in Additional file 2). An explanation of terms used in the survey is provided in Table 1. The survey included six profile questions relating to the respondent’s institution location, main research area and number of publications developed in collaboration with GSK Vaccines and independently. Of the 44 questions relating to publication practices, 37 had a closed format, and responders indicated the extent of their agreement with a series of statements (for example, ‘strongly agree’, ‘agree’, ‘neither agree nor disagree’, ‘disagree’ or ‘strongly disagree’) or a single response from a multiple-choice list. Several closed questions incorporated open follow-up questions to obtain further information on specific multiple-choice responses, such as ‘not clear at all’ or ‘other’. The remaining seven open-ended questions aimed to obtain opinions on topics such as ways to improve specific publication practices; responses to these questions are beyond the scope of this report because of their qualitative and heterogeneous nature. Four questions that aimed to obtain further information on user satisfaction with Datavision and, in order to focus solely on publication practices, six questions on congress-related activities were not included.

**Table 1:** **Terminology used in the survey**

Term	Explanation
Authorship questionnaire^a^ [25]	Questionnaire for identification of potential authors based on their level of contribution to a study and evaluation of their interest in authoring, taking public responsibility and critically reviewing and approving the final version, based on ICMJE criteria. Completed questionnaires are collected at the end of the active phase of the study, before study results are available.
Publication briefing meeting	First meeting with authors and technical support staff (as a minimum: the lead author, the corresponding author, publication writer and publications coordinator). Purpose is to provide the opportunity for authors to agree on the publication’s content (interpretation and presentation of results, references), authorship order, target journal, timelines and working practices.
Publication outline	Skeletal presentation of the text that is circulated to all authors as a basis for discussions of the structure and content of the publication.
Publication steering committee (PSC) [1]	Small working group of individuals that may include members of the study steering committee and protocol development team, investigators and other individuals with expertise in the area and scientists of the sponsor company. Its aim is to endorse and recommend publication activities on behalf of the research study group. PSC membership does not automatically confer authorship.
Core writing team	Subgroup of authors who commit to taking the lead in the development of a publication. All authors review and provide input to the scientific content of the manuscript.

^a^See Additional file 1.

### Survey participants and conduct

All investigators and researchers who had authored at least one publication reporting GSK Vaccines-sponsored human subject research since 2007 were invited to participate. Around 1,300 external authors had worked with the company during this period and the aim was to achieve a minimum response rate of 15%.

An email was issued by GSK Vaccines to introduce Deloitte as project partner and to guarantee confidentiality of feedback. The survey was issued on 22 May 2012 by email, along with a cover letter that explained its purpose, the nature of the group receiving the survey, its approximate duration and contact details in case of questions or concerns. Participants were invited to complete the survey by clicking on a hyperlink in the email that directed them to the web-based survey. The survey was hosted on a dedicated website that allowed responses to feed directly into a survey software tool (SurveyMonkey™ Palo Alto, CA, USA); Data were kept confidential and stored in a global database; personal identifying information (including respondents’ email addresses) was excluded from survey and data analysis procedures.

Participants were asked to complete the survey within three weeks. After two weeks, a reminder was sent to those who had not completed the survey. A second reminder was planned to be sent out after four weeks to invite non-respondents to participate over an extra period of two weeks. However, as the response rate achieved at the end of four weeks was higher than the minimum threshold of 15% specified in the study proposal, the survey was closed on 22 June 2012. The results were summarized as a percentage for each answer rounded to one decimal place. The results discussed in this manuscript are based on this voluntary survey and did not involve any experimental research on human subjects. Hence, ethical approval and informed consent procedures were not applicable.

## Results

The survey was sent to 1,273 investigators and researchers, 415 of whom provided feedback, giving a response rate of 32.6%. The respondents were from Europe (38.8%), Asia/Oceania (20.5%), North America (16.1%), Latin America (15.2%), Africa (8.0%) and the Middle East (1.5%).

**Figure 1:**
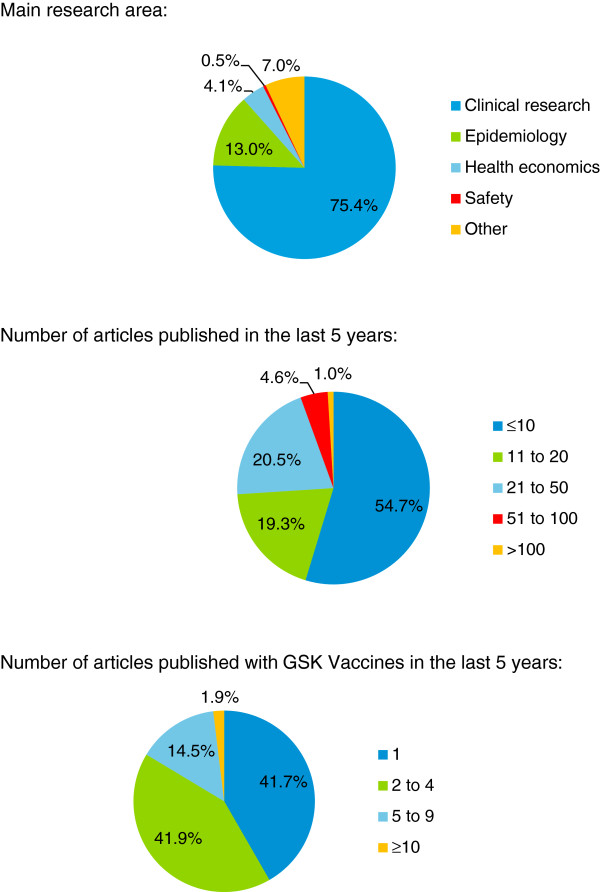
**Profile of participants: main research area and number of articles published in the last five years.** Percentages might not add up to 100% because of rounding.

Most respondents (n = 313) were clinical researchers (Figure 1). Since 2007, 54.7% (n = 227) had published 10 or fewer articles and 83.6% (n = 347) had published four or fewer articles in collaboration with GSK Vaccines (Figure 1).

### GSK Vaccines’ publications practices

#### Authorship questionnaire

**Figure 2:**
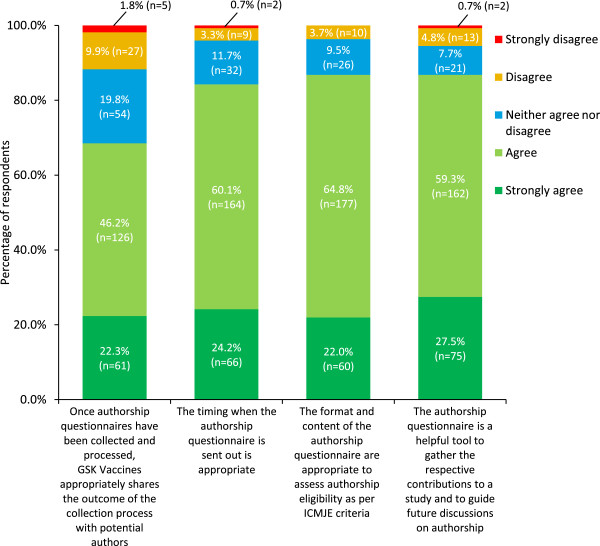
**Experience of the GSK Vaccines authorship questionnaire (based on ICMJE criteria).** Answers to the question ‘How much do you agree/disagree with the following statements?’ (n = 273).

Since 2011, GSK Vaccines has employed an authorship questionnaire (see Additional file 1) [25]. Of 273 respondents who had previously completed an authorship questionnaire, most agreed that its format and content is appropriate to assess eligibility for authorship according to ICMJE criteria, that it is a helpful tool for collecting information on authors’ contributions and guiding discussions on authorship, and that the timing of issuing the questionnaire (at the end of the active phase of the study, before study results are available) was appropriate (Figure 2). Over two-thirds of respondents agreed that the company communicates the outcome of the authorship process with authors appropriately (Figure 2).

#### Publication briefing meetings and publication outline

At the start of publication writing, all potential authors (based on results from the authorship questionnaire) are invited to participate in a briefing meeting to agree content, journal selection and timelines, and to confirm authorship and lead authorship. Less than half of respondents (41.2%; n = 171) had taken part in a briefing meeting. Of those who had taken part, over three-quarters (77.8%; n = 133) agreed that this had provided the opportunity for taking a greater role in authorship, content and journal selection, with 4.7% (n = 8) in disagreement and the remainder (17.5%; n = 30) neither agreeing nor disagreeing.

After the briefing meeting, an outline of the publication is produced by either the lead author alone or with writing support from a professional medical writer, according to the lead author’s preference. The outline is used as a basis for discussions among authors of the content of the publication. Nearly all respondents (95.2%; n = 395) agreed that this step improves the quality of the first draft of a publication, with 1.2% (n = 5) disagreeing and 3.6% (n = 15) neither agreeing nor disagreeing.

#### Publication steering committees and core writing teams

For certain trials, such as an international multicenter study or a complex study with multiple end-points, a PSC of external and internal study researchers may be formed to oversee and produce articles and presentations from the trial, as outlined in the GPP2 guidelines [1]. Of the 74 respondents who had been a member of a PSC, 83.8% (n = 62) agreed that this had provided the opportunity for a greater role in the publications planning of larger or complex studies, with 5.4% (n = 4) in disagreement and the remainder (10.8%, n = 8) neither agreeing nor disagreeing. Two-thirds of respondents felt that appointments to a PSC are made in a transparent manner (66.2% (n = 49); 8.1% (n = 6) disagreed, 25.7% (n = 19) neither agreed nor disagreed) and that the rationale for a PSC decision on publication planning and/or proposal and authorship is well disclosed and justified (66.2% (n = 49); 8.1% (n = 6) disagreed, 25.7% (n = 19) neither agreed nor disagreed).

A core writing team composed of a small group of authors may also be formed to lead the development of manuscript content, although all authors must review and provide input to the paper. Of the 163 participants who had been part of a core writing team, nearly all (96.3%; n = 157) agreed that this was helpful for developing publications, with 0.6% (n = 1) disagreeing and 3.1% (n = 5) neither agreeing nor disagreeing.

#### Use of a web-based publication tracking tool

A publication tracking tool (Datavision), designed to improve efficiency and ensure traceability of each step of publication development, is used as an interface between the publication coordinator and authors. Respondents stated that the key advantages of this tool were access to an ‘overview of publication status and pending tasks’ (31.0%; 213 of 688 advantages selected; respondents could select more than one), provision of a ‘secure environment’ (26.0%; n = 179), its ease of use (24.7%; n = 170) and transparency among users (15.7%; n = 108). Nearly three-quarters (72.3%; n = 300) of 415 respondents were satisfied or very satisfied with the use of this tool (8.7% (n = 36) not satisfied, 19.0% (n = 79) somewhat satisfied), although not all were familiar with each function, with 27.7% (n = 115) not knowing how to access other authors’ comments.

#### Transparency and communication

The survey included questions that aimed to gain feedback on the initiatives introduced since the previous 2009 survey in relation to transparency and communication of publication development practices. A total of 170 participants had collaborated with GSK Vaccines on publications before 2009 (41.1% of 414 respondents). Most (81.4%; 118 of 145) felt that overall transparency had improved over recent years. Of 101 respondents who had been involved in PSCs, 81.2% (n = 82) agreed that the transparency of PSC procedures had improved.

**Figure 3:**
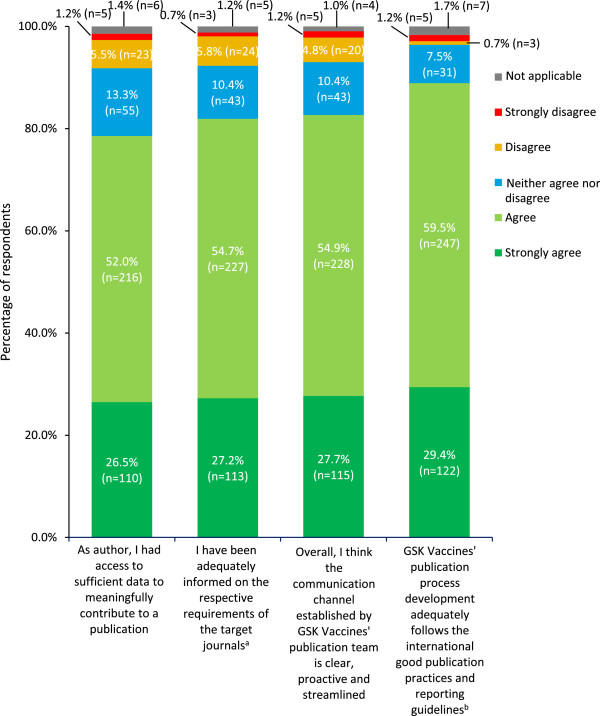
**Transparency of GSK Vaccines’ publication practices and decision-making.** Answers to the question ‘How much do you agree/disagree with the following statements?’ (n =415). ^a^Disclosure of medical writing support, conflicts of interest, contributorship and acknowledgement. ^b^Such as ICMJE, GPP2, CONSORT, STROBE, PRISMA, CHEERS and so on. Percentages might not add up to 100% because of rounding.

Most respondents felt that publication development practices adequately follow international good practice and reporting guidelines for publications and that communication was clear, proactive and streamlined (Figure 3). Over three-quarters of respondents agreed that, as authors, they had sufficient access to data to contribute meaningfully to the publication (Figure 3).

The majority of respondents agreed that they were kept well informed about publication development, including target journal requirements (Figure 3), submission and post-submission status (74.9% (n = 311) agreed, 6.5% (n = 27) disagreed) and publication development steps, timelines and target journal and congress selection (71.6% (n = 297) agreed, 9.6% (n = 40) disagreed). Around two-thirds of respondents (67.2% (n = 279)) felt well informed on upcoming publications (11.3% (n = 47) disagreed) and 58.3% (n = 242) on changes in authorship (12.5% (n = 52) disagreed). If an individual’s comment on a manuscript was not taken into account, 65.1% (n = 270) agreed that they were provided with a clear explanation as to why this was the case (6.3% (n = 26) disagreed). For each of these questions, the remainder of respondents replied ‘neither agree nor disagree’.

### General publication practices and standards

#### International Committee of Medical Journal Editors authorship criteria

**Figure 4:**
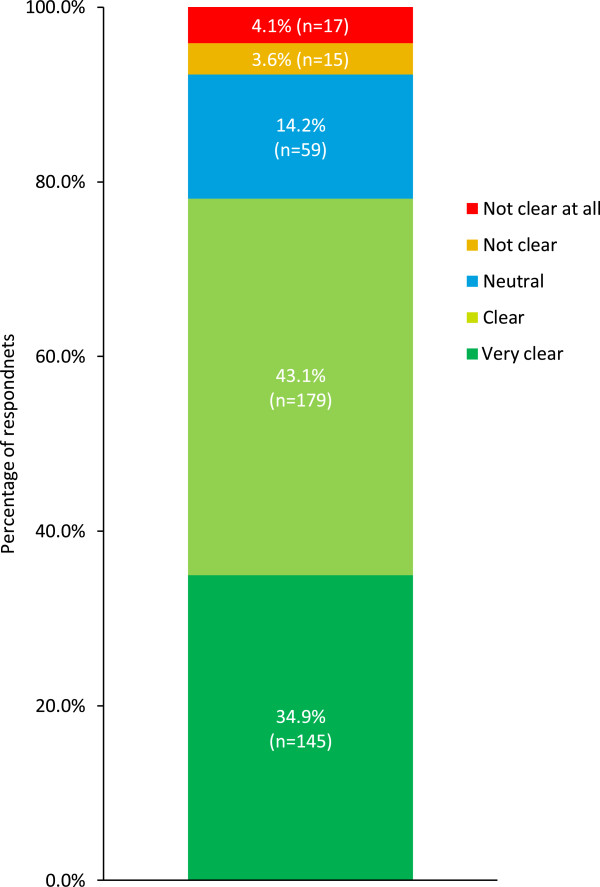
**ICMJE authorship criteria.** Answers to the question ‘How would you rate the clarity of the ICMJE criteria to evaluate authorship?’ (n = 415). Percentages might not add up to 100% because of rounding.

The ICMJE authorship criteria are followed by GSK. Those in place at the time of the survey were those introduced in 2008 [26] rather than the 2013 revision [16]. The 2008 ICMJE recommendations stated that authorship should be based on: 1) substantial contributions to conception and design, acquisition of data or analysis and interpretation of data; 2) drafting the article or revising it critically for important intellectual content; and 3) final approval of the version to be published. The ICMJE authorship criteria were regarded as ‘clear’ or ‘very clear’ to most participants, but 7.7% stated that the criteria were ‘not clear’ or ‘not clear at all’ (Figure 4).

#### Group authorship

Group authorship is sometimes used in order to attribute authorship (for example, of a multicenter trial) to a group name. Approximately three-quarters of respondents (75.9%; n = 315) stated that the concept of ‘group authorship’ is clear to them and 84.6% (341 of 403 respondents) indicated that they would feel comfortable being part of a group authorship. Those who were not comfortable with group authorship were invited in an open question to provide their reasoning; of the 54 comments received, 40.7% (n = 22) explained that group authorship had limited academic value, 22.2% (n = 12) felt that it is only appropriate for certain study designs, 18.5% (n = 10) stated that its concept is not clear and 18.5% (n = 10) cited practical issues.

#### Author agreements and timing of initial manuscript discussions

The GPP2 guidelines recommend that companies describe obligations for good publication practice in written publication agreements with authors and with members of writing groups or PSCs before the authors begin work [1]. This concept was popular with respondents; 81.7% (n = 339) stated that they would be in favor of introducing an ‘author agreement’, whereby authors and sponsors formally agree their respective responsibilities before work starts on publications.

Opinions were divided on the best time to start discussing a manuscript to be published in a peer-reviewed journal. The largest percentage (45.8%; n = 190) felt that discussions should begin at the start of protocol development or at the first investigators’ meeting for that study, while 26.5% (n = 110) felt they should start when the study’s statistical analysis report can be shared with investigators and researchers. The remaining respondents felt the appropriate timing was while the study is ongoing (13.0%; n = 54) or once the study results have been shared fully with investigators and researchers (14.7%; n = 61).

#### Professional medical writers and industry authorship

**Figure 5:**
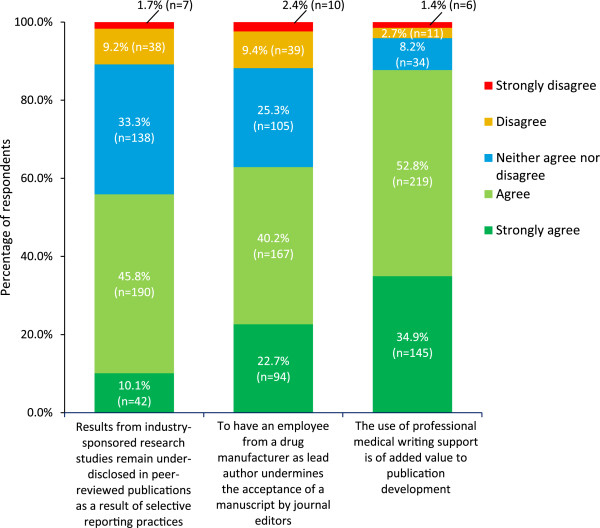
**Professional medical writers and industry authorship.** Answers to the question ‘How much do you agree/disagree with the following statements?’ (n = 415). Percentages might not add up to 100% because of rounding.

Professional medical writers work with authors to prepare publications and, in line with ICMJE authorship criteria [16], must be either named as authors or included in the acknowledgements section of manuscripts [23]. Most respondents agreed that the use of professional medical writing support can improve the publication under development, but that having an employee from a drug manufacturer as lead author reduces the possibility of acceptance of a manuscript by journal editors (Figure 5). Just over half of respondents felt that the results of industry-sponsored research remain under-disclosed in peer-reviewed publications because of selective reporting practices (Figure 5).

## Discussion

Many pharmaceutical companies have developed policies for the public disclosure of clinical research data to improve the transparency and credibility of industry-sponsored publications [10, 13, 21–24]. GSK has adopted publication practices based on international standards, and this survey gathered respondents’ opinions on the effectiveness of initiatives introduced following the first assessment in 2009. Other aims were to identify any remaining gaps in good publication practices and to gain insight into the understanding of publication standards by external authors.

The target population for this survey was investigators and researchers from different parts of the world who had previously authored publications in collaboration with GSK Vaccines. Therefore, respondents were likely to have been aware of best practices in publication and authorship criteria. This is a limitation of this study, as the results may not be representative of the perceptions of investigators and researchers who have not been involved with industry-sponsored research publications, or of the views of internal GSK Vaccines authors who did not take part in the surveys. The response rate for the survey was 32.6%, which might have led to response bias but is in line with average response rates for online surveys [27–29], and higher than that previously reported in an online survey of doctors in clinical practice [30].

Overall, the initiatives introduced since the 2009 survey appear to have been successful in improving transparency and communication relating to GSK publication development practices, as most respondents who had worked with GSK Vaccines before 2009 stated that overall transparency had improved, as had the transparency of PSC processes. However, there was some evidence that communication could be improved during publication development, particularly with regard to future publications, changes in authorship and reasons for not including authors’ suggested changes to content. More than 80% of participants felt that GSK publication practices adequately follow international good publication practice guidelines and that communication was clear and proactive. However, transparency could be improved further as about one in five respondents did not agree that they had sufficient access to data to meaningfully contribute to a publication. Authorship implies responsibility and accountability for published work [16], and recommendations and guidelines highlight the importance of ensuring that authors have access to the full dataset from the study, ideally before publication writing begins [1, 13]. Currently, GSK provides authors with statistical reports, followed by the full study report once available, and raw study data are provided upon request. In response to developments in the GPP field, the company is in the process of introducing new procedures, whereby authors will be asked to certify at the completion of a manuscript that they had the opportunity to access the analyzed data described in the publication and that the manuscript’s content is fair, accurate and balanced. An auditable procedure will be put in place to ensure that the full study report is shared before publications are started. GSK has also made commitments regarding public sharing of its research data [23], including the public disclosure of result summaries within specified periods of time from study completion, public availability of full study reports through a clinical trials register and access to anonymized patient-level data upon request [31].

In the present survey, 78% of respondents stated that the ICMJE criteria for authorship are clear to them. This high level of understanding is likely to have been due to the fact that all respondents had been involved with the company’s publication practices, and thus had been exposed to the measures put in place to clarify guidelines. Similar measures are not always in place, not only for industry-sponsored publications, but also for publicly-funded or academic reports [10, 12]. In a survey of 295 healthcare professionals, fewer than 60% were aware of ICMJE authorship criteria [32], and in a smaller survey of non-industry authors, approximately 40% of respondents were unaware of the ICMJE guidelines [33]. However, in our survey, one in five respondents did not agree that the ICMJE authorship criteria were clear. This suggests that not only should the means of communicating this information be examined, but that the clarity of the ICMJE authorship criteria to potential authors should be investigated further. The results of a recent survey conducted in Norway suggest this is particularly important for less experienced researchers [34]. For example, the term ‘substantial contributions’ included in the first ICMJE criterion can have different meanings to different individuals, as can the concept of accountability now included in the fourth criterion of the 2013 recommendations [16]. The guidelines also suggest that all individuals who meet the first criterion should have the opportunity to participate in the review, drafting and final approval of the manuscript. This could be extremely difficult to achieve in a reasonable timeframe for trials that involve a very large number of study investigators. Issues associated with authorship have been highlighted by the Medical Publishing Insights and Practices initiative, which has developed supplemental guidance to help authors set common rules for authorship early in a trial [35].

The latest ICMJE guidelines also acknowledge that, increasingly, authorship of multicenter trials is attributed to a group, and state that all members of the group who are named as authors should fully meet the ICMJE criteria for authorship [16]. In the 2012 survey, around one-quarter of respondents stated that the concept of group authorship is not clear. The updated guidance on group authorship and contributorship issued by the ICMJE should help improve the perception and understanding of this concept in industry-sponsored research, although not all journals adhere to these recommendations. Also, most journals do not provide practical advice on group authorship in their instructions for authors [36].

The 2009 survey revealed a lack of clarity about the rationale for authorship decisions. To address this, and to be consistent with best practice guidelines, the company developed an authorship questionnaire and systematically sends it out to investigators who were involved in a study and potentially qualify for authorship according to the first ICMJE authorship criterion [25]. Feedback from the current survey was encouraging, as most respondents considered the questionnaire to be a useful tool and judged its content to be appropriate for its purpose. This suggests that other companies should consider adopting a similar procedure. However, fewer than 70% of respondents agreed that the outcomes of the authorship questionnaires were shared sufficiently, and fewer than 60% felt well informed on changes in authorship (around 30% had a neutral opinion), indicating that communication on authorship determination and alterations can be improved.

Over 60% of respondents considered that the inclusion of an industry scientist (who fully meets ICMJE authorship criteria) as lead author reduces the likelihood of acceptance for publication by a journal. There is a lack of harmonized best practice guidance when it comes to authorship order and this survey result suggests that negative perceptions of industry-sponsored research could influence authorship practices. This is consistent with concerns raised by the editors and readers of medical journals in relation to industry-sponsored studies [37–42]. Also, results from the survey of 295 healthcare professionals revealed two-thirds of respondents were concerned about pharmaceutical employee involvement in manuscript preparation as authors or reviewers, even if disclosed [43].

Respondents were largely in favor of introducing a formalized ‘author agreement’ on responsibilities before starting work on a publication. This has since been adopted by GSK Vaccines, bringing the company’s policies further in line with GPP2 guidelines [1]. The most frequently expressed opinion on the best time to start discussing a manuscript also emphasized an early start, at protocol development or at the first study investigators’ meeting, which usually takes place after the protocol is finalized and the study has been approved, but before subject recruitment begins. Paradoxically, despite this expressed opinion, this has been difficult to achieve in the past, with the majority of potential authors preferring to start manuscript discussions when results become available, suggesting a change in mindset is needed to encourage discussions in the early stage of trials.

For the development of publication content, like other pharmaceutical companies, GSK Vaccines employs standardized procedures and tools for each step, including briefing meetings, publication outlines, PSCs and core writing teams, supported by access to a web-based file-sharing tool. Briefing meetings provide an opportunity for face-to-face or teleconference discussions, not only about the content of the manuscript, but to engage authors in the decision-making process on other aspects including authorship order, target journal and submission timelines. Of those who had taken part in a briefing meeting, more than three-quarters felt this to be of added value for their role in the publication development. The publication outline, produced as an outcome of the briefing meeting, was viewed by nearly all respondents as a valid step which improves the quality of the first draft of the manuscript.

A PSC may be formed at an early stage to oversee publications from a trial, as described in the GPP2 guidelines [1]. Others have reported that PSCs enhance publication development in terms of better involvement of external authors [44]. In our survey, participation in a PSC was considered to be helpful in providing the opportunity for taking a greater role in the planning of publications. However, improved transparency on PSC membership and the decision-making processes is needed, as only two-thirds of respondents felt that the appointment process to join a PSC is transparent and that the rationale of PSC decisions is well disclosed and justified. Nearly all respondents agreed that implementation of a core writing team, involving a small group of authors, is beneficial for manuscript development.

The web-based file-sharing tool allows users to manage the secure circulation and review of each publication draft, and also allows the publication coordinator to check that authors have contributed to all stages and provided final approval. This tool was perceived as advantageous and most respondents were satisfied with its use. The functionality of this tool continues to evolve to address user requirements, such as the need to access co-authors’ comments, which was highlighted by the results of the 2009 survey. However, as over a quarter of respondents to the 2012 survey did not know how to access this specific function, it is clearly important to ensure that users know how to use this tool.

In the present survey, nearly 90% of respondents agreed that the use of professional medical writing support can add value in the development of a manuscript. This was in broad agreement with other surveys that have examined perceptions of professional medical writers. In a survey of 204 healthcare professionals with previous publications, 68% said they would agree to be an author of a publication developed with medical writing assistance [43]. Further, a survey of 76 authors reported that 83% felt it was acceptable to receive assistance and 84% valued the assistance provided by professional medical writers [45]. However, of 295 healthcare professionals, 30% stated that they would trust a peer-reviewed publication less if medical writers were involved and only 14% would trust it more [43], which is consistent with reports of concern in the medical literature about the involvement of professional medical writers [9, 46, 47]. This may be partly due to the frequent description of professional medical writing assistance as ghostwriting [48]. Professional medical writers work with authors to prepare publications while ensuring authors control and direct writing, and that disclosures of funding, potential conflicts of interest and acknowledgment of contributions are made [1, 49]. In contrast, ghostwriters are individuals who analyze data and/or write manuscripts, but are not acknowledged in the publication [9, 10, 47]. In line with other pharmaceutical companies [10, 21, 24], GSK’s policy is to name professional medical writers in the article either as authors when their contribution meets authorship criteria, or by description of their contribution within the acknowledgements section [23]. However, the level of concern surrounding professional medical writer involvement, and industry-sponsored research in general, suggests that further efforts are needed to highlight the distinction between writing and editing contributorship and authorship. This may include standardization of the definition of ghostwriting, as well as an open debate of the ethics and value of professional medical writing assistance [10, 13, 48, 50].

## Conclusions

The pharmaceutical industry has acted to counter criticisms of publication practices for industry-sponsored research by incorporating internationally-recognized recommendations and guidelines into their policies and standard practices, but opportunities remain for additional improvement [13]. Prompted by the results of a clinical investigator survey conducted in 2009, GSK Vaccines made changes to its publication practices to improve transparency and communication with external authors. The results of this second survey conducted in 2012 suggest that these changes have been effective to a large extent, but that further improvements should be made to enhance good publication practices.

Overall, the usefulness and suitability of the company’s publication practices were acknowledged by the majority of external authors responding to this survey. Authors were in favor of initiating discussions about publications, including authorship, at an early stage and with the introduction of a formal ‘author agreement’ from the outset. There was some evidence that external authors would benefit from improved transparency and communication from GSK Vaccines in relation to the outcomes of the authorship questionnaire, changes in authorship, publication progress and future publications. Although PSCs are recognized to be valuable, there was sometimes a lack of clarity in PSC procedures and outcomes. The need for improved communication in relation to outcomes of authorship selection and on the practical use of the web-based publication tracking tool was also highlighted, as well as the need for sufficient access to relevant data for all authors. The company’s publication practices are being strengthened to ensure data access that permits meaningful contributions by all authors to each publication. Moreover, the results suggest further clarification of best practices for authorship should be provided in international recommendations and in journals’ instructions to authors.

The survey results confirm the need for publication practices and tools that ensure effective and timely communication, and transparent processes for authorship and decision-making. The identified gaps in good publication practice will help to guide further improvements to the company’s policies on publication development.

## Authors’ information

Present institutional addresses for TM: KU Leuven, Leuven, Belgium, and UCB Pharma, Anderlecht, Belgium.

## Electronic supplementary material

Additional file 1: **Authorship questionnaire (authorship_questionnaire.pdf; questionnaire sent to authors to determine eligibility for authorship).** (PDF 71 KB)

Additional file 2: **Survey questionnaire (survey_questionnaire.pdf; survey questions answered by investigators/researchers).** (PDF 890 KB)

## Abbreviations

GPP: Good publication practice
GSK: GlaxoSmithKline
ICMJE: International Committee of Medical Journal Editors
PSC: Publication steering committee.
