# Supplementary material for: Publication practices and standards: recommendations from GSK Vaccines’ author survey
Source: Trials. 2014 Nov 18;15:446. doi: 10.1186/1745-6215-15-446 (PMC4247206; doi:10.1186/1745-6215-15-446)
Supplement: Supplementary file 1 — Additional file 1: Authorship questionnaire (authorship_questionnaire.pdf; questionnaire sent to authors to determine eligibility for authorship). (PDF 71 KB) [file 13063_2014_2308_MOESM1_ESM.pdf]

STUDY NUMBER:

AUTHORSHIP QUESTIONNAIRE

Section 1. Identifying information

NAME:

FUNCTION:

ACADEMIC DEGREE(S):

CONTACT DETAILS:

AFFILIATION*:

DEPARTMENT:

POSTAL ADDRESS:

ZIP/POSTAL CODE: CITY: COUNTRY: Please choose as appropriate

TELEPHONE:

FAX:

* *For GSK in-/out-sourced contractors, please indicate your CRO/freelance affiliations*

1. Your role in the study: Please choose as appropriate

Proposed Publication Plan

ICMJE : CRITERIA FOR AUTHORSHIP

An author is an individual who has made substantial intellectual contributions to a publication and should have participated sufficiently in the work to meet all of the following ICMJE criteria (ICMJE; http://www.icmje.org):

1. **Substantial contributions to conception and design, acquisition of data, or analysis and interpretation of data**
2. **Drafting the article or revising it critically for important intellectual content**
3. **Final approval of the version to be published**
4. **Agreement to be accountable for all aspects of the work in ensuring that questions related to the accuracy or integrity of any part of the work are appropriately investigated and resolved.**

Section 2. Authorship

	YES	NO
2. Are you interested in taking an active role as an author?	◯	◯
3. If yes, to which publication(s) of the proposed publication plan?		
4. Would you like to be a member of the writing committee? (if applicable)	◯	◯
5. Would you like to be lead author?	◯	◯
6. If you DO NOT wish to be an author, would you like your contribution to this project to be acknowledged in the publication?	◯	◯

WRITING COMMITTEE

The writing committee includes a group of authors who commit to take the lead on the publication's development. The lead author and the publication writing committee are responsible for providing proposals for implementation of comments and for resolving potential conflicting feedbacks from authors. For transparency reasons, it is increasingly common practice to disclose the writing committee membership in the publication.

AUTHORSHIP DEFINITIONS

Lead author: The lead author takes the lead for writing and managing the article (GGP2; http://www.gpp-guidelines.org/)

Corresponding author: The corresponding author functions as the primary contact between the journal and the other authors. The corresponding author does not need to be the lead author, but should be selected for his/her ability to help co-ordinate the review and revision process (MPIP; http://www.ismpp.org/initiatives/Files/MPIP_Authors_Submission_Toolkit.pdf).

Section 3. Your expertise

7. Please detail your relevant expertise regarding the content of the publications (to give a better insight into your expertise and publication background, please feel free to provide a current copy of your CV in English)

RESPONSIBILITIES OF AUTHORS

The scientific publications group at GSK can provide support for developing the publication, however, it is the specific responsibility of the authors to have an active role in all of the following: (GGP2; http://www.gpp-guidelines.org/)

- **Collaborate in the development of the manuscript outline**
- **Critically review and comment on successive drafts**
- **Approve the final draft before submission and any revisions resulting from editorial comments**

Please note that according to the guidelines on Good Publication Practice (GPP2), "no honorariums are paid for authorship of peer reviewed articles or presentations".

Investigators/experts not willing to be authors will have a chance to be acknowledged in the publication.

Section 4. Congress presentation

YES NO

8. Are you interested in being a presenting author of the results at a scientific congress? (if applicable) ◯ ◯

A presenting author should meet the qualifications for authorship. On top of these, it is preferred that the person has good presentation skills and is fluent in the language of the presentation.

Section 5. Publication writing

9. Are you interested in writing the publication? YES ◯ NO ◯

Would you need the support of a medical writer?

SUPPORT FOR AUTHORS

Authors must contribute to the writing/content development of the publication and are responsible for content. Although GSK can provide writing support*, authors are welcome to draft the manuscript themselves and implement the comments of all authors received during review according the GSK processes. The GSK scientific publication group will ensure the drafts are circulated to all authors and sponsor reviewers and comments are provided to authors for implementation.

***Writing support: is the technical writing support provided by a professional medical writer. Professional medical writers should ensure that authors control and direct writing. Particular care is taken to ensure appropriate acknowledgement of the contributions made by medical writers and to describe their funding (ICMJE; http://www.icmje.org). (Section taken literally from ICMJE)**

STUDY NUMBER:

Section 6. Your contribution to study

10. Please indicate in the table below your contributions to the study. These will help to determine eligibility for authorship or acknowledgement, according to the ICMJE criteria

Contribution to Study	YES	NO	Comment (if applicable)
Conception/design/planning of the study	○	○	
Acquisition/assembling of data	○	○	
Contribution to:			
Center coordination	○	○	
Data extraction	○	○	
Quality check	○	○	
Performing or supervising the analysis	○	○	
Interpretation of results	○	○	
Provision of study materials or subjects	○	○	
Provision of statistical expertise	○	○	
Collection of Data	○	○	
Acquisition of funding	○	○	
Choice/recruitment of centers/recruitment of investigators	○	○	
Administrative, technical or logistic support	○	○	
Laboratory/serology testing	○	○	
Supervision of the study/research group	○	○	
Other (please specify):	○	○	

AGREEMENT

I agree that my responses to this questionnaire will be used by GlaxoSmithKline Biologicals SA and its affiliated companies for the purpose of planning, preparing and publishing scientific publications arising out of the Study and will be archived for documentation purposes in GlaxoSmithKline Biologicals SA repository.
