# Supplementary material for: Publication practices and standards: recommendations from GSK Vaccines’ author survey
Source: Trials. 2014 Nov 18;15:446. doi: 10.1186/1745-6215-15-446 (PMC4247206; doi:10.1186/1745-6215-15-446)
Supplement: Supplementary file 2 — Additional file 2: Survey questionnaire (survey_questionnaire.pdf; survey questions answered by investigators/researchers). (PDF 890 KB) [file 13063_2014_2308_MOESM2_ESM.pdf]

E-MAIL: INTRODUCTION TO THE SURVEY AND INVITATION TO PARTICIPATE

Deloitte.

Dear Doctor ....,

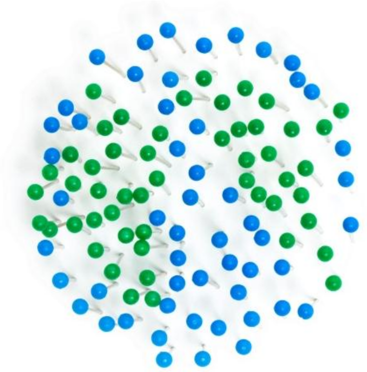

As you have recently been informed by GlaxoSmithKline Biologicals, Deloitte has been selected to conduct a **survey to collect feedback on GlaxoSmithKline Biologicals' current publication process**.

This survey is being circulated to all investigators/researchers collaborating with GlaxoSmithKline Biologicals on publications in the world.

GlaxoSmithKline Biologicals has implemented a number of initiatives over the past five years to make its publication process more efficient. This survey is meant to collect your views on the progress achieved and to identify possible areas for further improvement.

We value your feedback and would therefore be grateful if you would agree to participate in this survey.
May we kindly remind you that the **closing date is June 8th 2012**.

Please click here to access the survey. It will take only 15 to 20 minutes of your time.

Please note that Deloitte is acting as an independent third party: your individual feedback will remain entirely confidential.

Thank you for your collaboration
The Deloitte team

Would you have any question or concern, please contact us by email at the following address:
besurveypublication@deloitte.com

Deloitte refers to one or more Deloitte Touche Tohmatsu, a Swiss Verein, and its network of member firms, each of which is a legally separate and independent entity. Please see www.deloitte.com/about for a detailed description of the legal structure of Deloitte Touche Tohmatsu and its member firms.

Home | RSS | **Add** Deloitte **as safe sender**

Survey on the publication process supported by GlaxoSmithKline Biologicals

1. Survey Introduction

As you have recently been informed by GlaxoSmithKline Biologicals, Deloitte has been commissioned to conduct a survey to collect feedback on GlaxoSmithKline Biologicals' current publication process.

GlaxoSmithKline Biologicals has implemented a number of initiatives over the past five years to make its publication process more transparent and efficient. This survey is meant to collect your views on the progress achieved and to identify possible areas for further improvement.

This survey will only take 15-20 minutes of your time to complete and its results will be processed completely anonymously.

To start survey, please click NEXT.

Survey on the publication process supported by GlaxoSmithKline Biologicals

2. Your profile

Your information will remain entirely confidential.

*1. Your contact information (email):

*2. Please indicate the region of your host institution:

- Europe
- Asia / Oceania
- Northern America
- Latin America
- Middle East
- Africa

*3. How many scientific articles have you published over the past 5 years?

Number of articles

*4. How many scientific articles have you published in collaboration with GlaxoSmithKlineBiologicals over the past 5 years?

- 1
- 2 - 4
- 5 - 9
- > =10

*5. Please indicate your main research area:

- Clinical research
- Epidemiology
- Health Economics
- Safety
- Other

Other (please specify)

6. Please indicate the area where you have the largest scientific publication experience in collaboration with GlaxoSmithKline Biologicals:

Largest experience

Other (please specify)

Survey on the publication process supported by GlaxoSmithKline Biologicals

3. Publication process - Authorship

The International Committee of Medical Journal Editors (ICMJE)is a small working group of general medical journals, not an open-membership organization - created the Uniform Requirements primarily to help authors and editors in their mutual task of creating and distributing accurate, clear, easily accessible reports of biomedical studies.

http://www.icmje.org/

*7. How would you rate the clarity of the ICMJE criteria to evaluate authorship?

	Very clear	4	3	2	Not clear at all
ICMJE criteria	○	○	○	○	○

If not applicable/not sure, please comment here.

8. Does GlaxoSmithKline Biologicals provide you sufficient guidance/information on your role and responsibility as author?

○ Yes

○ No

Survey on the publication process supported by GlaxoSmithKline Biologicals

4. Publication process - Authorship (continued)

Since 2009, GlaxoSmithKline Biologicals has been implementing "authorship questionnaires" to ascertain and document:

a) compliance with the ICMJE criteria for authorship,
b) the respective past/ongoing contributions to the study, and
c) the interest/availability to contribute to a future scientific article.

*9. Have you ever received the "Authorship Questionnaire" from GlaxoSmithKline Biologicals?

- Yes
- No

Survey on the publication process supported by GlaxoSmithKline Biologicals

5. Publication process - Authorship (continued)

*10. How much do you agree or disagree with the following statements?

	Strongly agree	Agree	Neither agree or disagree	Disagree	Strongly disagree
The "authorship questionnaire" is a helpful tool to gather the respective contributions to a study and to guide the future discussion on authorship.	○	○	○	○	○
Format and content of the authorship questionnaire are appropriate to assess authorship eligibility as per the ICMJE criteria.	○	○	○	○	○
The timing when the "authorship questionnaire" is sent out is appropriate.	○	○	○	○	○
Once all "authorship questionnaires" have been collected and processed, GlaxoSmithKline Biologicals appropriately shares the outcome of the collection process with all potential authors.	○	○	○	○	○

Survey on the publication process supported by GlaxoSmithKline Biologicals

6. Publication process Authorship (continued)

*11. Is the concept of "group authorship" clear to you?

- Yes
- No

12. Would you feel comfortable with the idea of being part of a "group authorship" for a study?

- Yes
- No

If no, could you explain?

*13. Currently, GlaxoSmithKline Biologicals does not implement any form of "author agreement", whereby authors and sponsor formally agree on their respective responsibilities before the publication process starts. Would you be in favour of such an agreement in future?

- Yes
- No

14. Would you like to suggest any alternative to "authorship questionnaires"?

Survey on the publication process supported by GlaxoSmithKline Biologicals

7. Publication process- Briefing meetings

A "briefing meeting" is now systematically organised with at least the lead author at the start of the publication process to agree on: the publication content, authorship, journal selection and timelines.

* 15. Have you ever participated in a "briefing meeting"?

- Yes
- No

Survey on the publication process supported by GlaxoSmithKline Biologicals

8. Publication process- Briefing meeting (continued)

*16. How much do you agree or disagree with the following statements?

	Strongly agree	Agree	Neither agree or disagree	Disagree	Strongly disagree
The systematic organisation of "briefing meetings" has granted me a greater role in authorship, content and journal selection discussions.	○	○	○	○	○
"Briefing meetings" are efficiently organised by GlaxoSmithKline Biologicals.	○	○	○	○	○
I have had the opportunity to provide enough input for the selection and order of authors.	○	○	○	○	○
My viewpoint on the journals selection was taken into account.	○	○	○	○	○

17. Would you have any suggestion to further improve the organisation of “briefing meetings"?

Survey on the publication process supported by GlaxoSmithKline Biologicals

9. Publication process - Publication Steering Committees

In the case of international multi-centre studies, "Publication Steering Committees" (PSC) may be organised to endorse: the publication plan, authorship, journal selection and timelines.
A PSC comprises a limited number of members with representation of investigators/researchers and GlaxoSmithKline Biologicals scientific/medical staff. Several publications, resulting from a single study, are usually discussed by the PSC.

* 18. Have you ever been a member of a PSC for a given study?

- Yes
- No

10. Publication process - Publication Steering Committees (continued)

*19. How much do you agree or disagree with the following statements?

	Strongly agree	Agree	Neither agree or disagree	Disagree	Strongly disagree
The establishment of "Publication Steering Committees" has granted a greater role to investigators/researchers in the publication planning of large studies.					
The appointment process to join a PSC is made in a transparent manner.					
PSC meetings are usually well prepared and facilitated by the publication managers (i.e. agenda, presentation, minutes).					
The rationale of a PSC decision on publication planning/proposal and authorship is always well disclosed and qualified.					

20. Would you have any suggestion to further improve the management of PSCs?

11. Publication process - Core-writing Teams

The core-writing team is a small group of authors leading the development of the content of a manuscript with large authorship.

*21. Have you ever been a member of a core-writing team?

- Yes
- No

22. How much do you agree or disagree with the following statements?

	Strongly agree	Agree	Neither agree or disagree	Disagree	Strongly disagree
A core-writing team is of added value for the development of a manuscript.					
If a publication manager is involved in a core-writing team, his/her role and responsibilities are clearly defined and agreed upfront.					

Survey on the publication process supported by GlaxoSmithKline Biologicals

12. Publication process - Content of the scientific publication

*23. How much do you agree or disagree with the following statements?

	Strongly agree	Agree	Neither agree or disagree	Disagree	Strongly disagree
The development of an outline improves the quality of the first draft of a publication.	○	○	○	○	○
I had the opportunity to provide input on the content of the publication at early stage.	○	○	○	○	○
My feedback on the manuscript content was taken into account.	○	○	○	○	○
If my feedback was not taken into account, clear explanations were provided.	○	○	○	○	○
The current publication process is well-designed and offers sufficient time for me to provide input.	○	○	○	○	○

Survey on the publication process supported by GlaxoSmithKline Biologicals

13. Publication process - Scientific congress related activities

*24. Have you been involved in plenary session activities at scientific congresses in collaboration with GlaxoSmithKline Biologicals?

- Yes
- No

Survey on the publication process supported by GlaxoSmithKline Biologicals

14. Publication process - Scientific congress related activities (continued)

*25. How much do you agree or disagree the following statements?

	Strongly agree	Agree	Neither agree or disagree	Disagree	Strongly disagree
I am sufficiently involved in the planning and decision around congress activities (i.e. abstracts, poster and presentation).	◯	◯	◯	◯	◯
The selection of the presenting author(s) is appropriate and transparent.	◯	◯	◯	◯	◯

*26. Did you interact with an external agency (hired by GlaxoSmithKline Biologicals) on the preparation of activities related to scientific congresses?

◯ Yes

◯ No

27. If yes, how satisfied are you with the interactions you had with the external agencies?

◯ Very satisfied ◯ Satisfied ◯ Somehow Satisfied ◯ Not Satisfied ◯ Not Satisfied at all

*28. Overall, how satisfied are you with the congress related activities, as managed by GlaxoSmithKline Biologicals?

◯ Very satisfied ◯ Satisfied ◯ Somehow Satisfied ◯ Not Satisfied ◯ Not Satisfied at all

29. Would you have any suggestion to further improve the organisation of congress related activities?

Survey on the publication process supported by GlaxoSmithKline Biologicals

15. Publication process - Transparency of process and decision making

*30. In your opinion, when is the ideal time to start discussing a manuscript to be published in a peer-reviewed journal (title, authorship, and journal)?

- At the start of protocol development
- At first investigators meeting
- Whilst the study is ongoing
- As the study results are about to be made available
- Once the study results have been shared fully

*31. How much do you agree or disagree with the following statements?

	Strongly agree	Agree	Neither agree or disagree	Disagree	Strongly disagree	Not applicable
GlaxoSmithKline Biologicals' publication process development adequately follows the international publication good practices and reporting guidelines (such as ICMJE, GPP2, CONSORT, ISPOR, STROBE, PRISMA etc).						
Overall, I think the communication channel established by GlaxoSmithKline Biologicals' publication team is clear, proactive and streamlined.						
I have been adequately informed on the respective requirements of the target journals (i.e. disclosure of medical writing support, conflicts of interest, contributor-ship, and acknowledgments).						
As author, I had access to sufficient data to meaningfully contribute to a publication.						

Survey on the publication process supported by GlaxoSmithKline Biologicals

*32. I am well informed on

	Strongly agree	Agree	Neither agree or disagree	Disagree	Strongly disagree
• upcoming publications	○	○	○	○	○
• publication development steps and timelines	○	○	○	○	○
• target journal(s) /congress selected	○	○	○	○	○
• changes in authorship	○	○	○	○	○
• submission and post-submission status (submission, editors' decision, publication, e-publication)	○	○	○	○	○

Survey on the publication process supported by GlaxoSmithKline Biologicals

16. Publication process - Datavision

Datavision is a secure web-based interface between GlaxoSmithKline Biologicals and authors to develop a publication.

Before first use, an email is sent out by GlaxoSmithKline Biologicals with log-in details, instructions and user-guide.

* 33. Do you find the user-guide sent by GlaxoSmithKline Biologicals provides sufficient and timely information to use Datavision?

- Yes
- No

* 34. Do you know how to access other co-authors' comments via Datavision?

- Yes
- No

35. If yes, do you usually take time to review the comments made by other co-authors in Datavision?

Always | Often | Sometimes | Hardly ever | Never

* 36. Datavision provides a list of outstanding tasks assigned to you as co-author. How often do you check this list?

Always | Often | Sometimes | Hardly ever | Never

Survey on the publication process supported by GlaxoSmithKline Biologicals

17. Publication process - Datavision (continued)

*37. How often have you experienced issues when accessing Datavision?

- Always
- Often
- Sometimes
- Hardly ever
- Never

38. If you have encountered issues, please specify: (multiple choice is possible)

- Log-in & password issues
- Document Uploading & downloading issues
- Access issue when travelling
- Unable to access via smart phone or Ipad
- Other

Other (please specify)

*39. In your opinion, what are the key advantages of Datavision?

- Easy to use
- Secure environment
- Overview of publication status and pending tasks
- Transparent
- Other

Other (please specify)

*40. Overall, how satisfied are you with the use of Datavision?

- Very satisfied
- Satisfied
- Somehow Satisfied
- Not Satisfied
- Not Satisfied at all

41. Would you have any suggestion to further improve the use of Datavision?

Survey on the publication process supported by GlaxoSmithKline Biologicals

18. Publication process - General perception on the publication of company spon...

*42. How much do you agree or disagree with the following statements?

	Strongly agree	Agree	Neither agree or disagree	Disagree	Strongly disagree
The use of professional medical writing support is of added-value to the publication development.					
To have an employee from a drug manufacturer as lead author undermines the acceptance of a manuscript by journal editors.					
Results from industry-sponsored research studies remain under-disclosed in peer-reviewed publications as a result of selective reporting practices.					

*43. In your opinion, who is the most appropriate person to be the corresponding author?

- First author/ lead author
- Senior author
- Any author
- Last author
- Co-author working at the industry sponsor
- Other

Other (please specify)

44. In your opinion, what are the main responsibilities of

- Lead author?
- Corresponding author?

Survey on the publication process supported by GlaxoSmithKline Biologicals

19. Your collaboration with GlaxoSmithKline Biologicals before 2009

In 2009, GlaxoSmithKline Biologicals publication team conducted a survey to receive feedback from investigators on the publication process. Based on the feedback received, GlaxoSmithKline Biologicals has implemented a number of initiatives to improve the publication process.

*45. Did you collaborate on any scientific publication with GlaxoSmithKline Biologicals before 2009?

- ○ Yes
- ○ No

Survey on the publication process supported by GlaxoSmithKline Biologicals

20. Your collaboration with GlaxoSmithKline Biologicals before 2009 (continued)

*46. Over the past a few years,

	Yes	No	Not applicable
if you have been involved in PSCs, do you feel that the PSC process has improved over that period of time?			
if you have been involved in scientific congresses in collaboration with GlaxoSmithKline Biologicals, do you feel that the management of congress activities has improved over that period of time?			
if you have been collaborating on scientific publications with GlaxoSmithKline Biologicals, do you feel that the overall transparency of the publication development has improved over that period?			
if you have been using Datavision software, do you feel its functionality has improved over that period of time? (e.g. log-in help, use instructions in first email etc)?			

Survey on the publication process supported by GlaxoSmithKline Biologicals

21. Overall satisfaction

This is the last page of this survey.

*47. Overall, are you satisfied with the interactions you have with GlaxoSmithKline Biologicals when working on scientific publications?

- Very satisfied
- Satisfied
- Somehow Satisfied
- Not Satisfied
- Not Satisfied at all

*48. Compared with the other pharmaceutical companies that you have been collaborating with, how would you rate the publication process managed by GlaxoSmithKline Biologicals?

- Top tier
- Middle tier
- Lower tier

49. Do you feel that any topic of importance has not been covered in this survey?
If so, please share with us any comment that you may have.

50. GlaxoSmithkline Biologicals' publication team would like to consult a few investigators on some specific aspects in a year's time (May 2013) – would you agree to participate in this future survey?
(NB: this short survey will take maximum 5 minutes to fill in)

- Yes
- No

Survey on the publication process supported by GlaxoSmithKline Biologicals

22. End of survey

This is the end of the survey, please click "SUBMIT" to send your answers.

THANK YOU FOR PARTICIPATING IN THIS SURVEY!
